# A new concept for temporal gating of synchrotron X-ray pulses

**DOI:** 10.1107/S1600577521000151

**Published:** 2021-02-05

**Authors:** D. Schmidt, R. Bauer, S. Chung, D. Novikov, M. Sander, J.-E. Pudell, M. Herzog, D. Pfuetzenreuter, J. Schwarzkopf, R. Chernikov, P. Gaal

**Affiliations:** a Institut für Nanostruktur- und Festkörperphysik, Universität Hamburg, Luruper Chaussee 149, 22761 Hamburg, Germany; b Deutsches Elektronen-Synchrotron (DESY), 22607 Hamburg, Germany; c Paul-Scherrer Institute, Forschungsstrasse 111, 5232 Villigen, Switzerland; d Institut für Physik und Astronomie, Universität Potsdam, 14476 Potsdam, Germany; e Leibniz-Institut für Kristallzüchtung, Max-Born-Strasse 2, 12489 Berlin, Germany; f Canadian Light Source Inc., 44 Innovation Boulevard, Saskatoon, Canada SK S7N 2V3

**Keywords:** synchrotron, time-resolved, thermal deformation, transient grating, pulse picking

## Abstract

A new concept for temporal gating of synchrotron X-ray pulses based on laser-induced thermal transient gratings is presented. First experimental tests of the concept yield a diffraction efficiency of 0.18%; however, the calculations indicate a theoretical efficiency and contrast of >30% and 10^−5^, respectively. The full efficiency of the pulse picker has not been reached yet due to a long-range thermal deformation of the sample after absorption of the excitation laser. This method can be implemented in a broad spectral range (100 eV to 20 keV) and is only minimally invasive to an existing setup.

## Abstract 2

A new concept for temporal gating of synchrotron X-ray pulses based on laser-induced thermal transient gratings is presented.

## 1. Introduction   

Passive optical elements, for example monochromators, mirrors or lenses, are used in almost all synchrotron and free-electron laser (FEL) beamlines to tailor the properties of the emitted beam to the requirements of a specific experiment. Without these components the facilities could not host the broad range of science applications as they do today (Hand, 2009 ▸; Waldrop, 2014 ▸; Weckert, 2015 ▸). With the advent of high-power radiation sources adaptive optics are being developed to enable dynamic optimization of the beam conditions (Stoupin *et al.*, 2010 ▸; Quintana *et al.*, 1995 ▸; Berman & Hart, 1991 ▸; Yashchuk *et al.*, 2015 ▸; Goto *et al.*, 2015 ▸). Today most parameters of the emitted pulses can be tuned, for example energy and bandwidth, beam divergence, focal size or even the time structure of the emitted pulse itself (Schoenlein *et al.*, 2000 ▸; Sander *et al.*, 2019 ▸). The remaining parameter that can only be controlled with large effort is the time structure of the emitted pulse train. At most beamlines it is determined by the filling pattern of electron bunches in the storage ring. To accommodate all user needs, synchrotrons provide different bunch patterns over the year (Jankowiak & Wüstefeld, 2013 ▸; see also https://www.esrf.eu/Accelerators/Operation/Modes, https://photon-science.desy.de/facilities/petra_iii/machine/parameters/index_eng.html). In consequence, not all experiments available at a facility can be offered at the same time and some applications, in particular time-resolved experiments, constantly face an unfavorable time structure. In view of current upgrade programs to fourth-generation storage rings, this problem is expected to become even more pressing (Schroer *et al.*, 2018 ▸).

Few solutions exist that allow the time structure of the X-ray pulse pattern to be changed. The most reliable among them are mechanical choppers (LeGrand *et al.*, 1989 ▸; Wulff *et al.*, 2002 ▸; Gembicky & Coppens, 2007 ▸; Meents *et al.*, 2009 ▸; Kudo *et al.*, 2009 ▸; Ito *et al.*, 2009 ▸; Husheer *et al.*, 2012 ▸; Plogmaker *et al.*, 2012 ▸; Wang *et al.*, 2015 ▸; Förster *et al.*, 2015 ▸). Besides many technical challenges, their main disadvantage is the low flexibility of the devices which generally prevents a transfer to another setup. Other approaches employ rotating crystals (McPherson *et al.*, 2002 ▸) or piezoelectric crystals (Grigoriev *et al.*, 2006 ▸) to deflect individual X-ray bunches from the incident pulse train. Recently, this idea was successfully realized by oscillating micro-electromechanical structures (Mukhopadhyay *et al.*, 2015 ▸; Chen *et al.*, 2019 ▸). This method works only with monochromatic X-ray pulses and is generally limited to specific pre-selected pulse repetition rates. An undulator-based bunch kicker capable of isolating single bunches from a hybrid filling pattern was demonstrated at BESSY II (Holldack *et al.*, 2014 ▸). Finally, a promising concept relies on propagating surface acoustic waves (SAWs) which modulate the diffraction efficiency of a substrate Bragg peak (Roshchupkin *et al.*, 2003 ▸; Vadilonga *et al.*, 2017*a* ▸). Due to the electronic control of the SAWs, this method provides the highest flexibility and is least invasive to an existing setup (Tucoulou *et al.*, 1997 ▸; Vadilonga *et al.*, 2017*b* ▸). However, since it relies on Bragg diffraction from a crystalline substrate, it is limited to monochromatic hard X-rays.

In this work we present a new approach to control the time structure of an X-ray pulse train emitted by a synchrotron storage ring. Our method employs laser-induced thermal surface distortions with lateral periodicity. A grazing-incidence X-ray pulse is diffracted from the thermal transient grating (TG) away from the specular reflection and can be separated with an aperture or an analyzer crystal. The dynamics of the thermal grating can be controlled by the optical excitation (Pudell *et al.*, 2019 ▸), and temporal gating with opening times of 50 ps were demonstrated (Sander *et al.*, 2017*a* ▸) with 1 ps optical pulses for the excitation of the TG. The theoretical limit of the diffraction efficiency is 33% (Sander *et al.*, 2017*b* ▸). Realizing such high diffraction efficiency from the TG requires the generation of thermal surface gratings with amplitudes of few nanometres. Here we present surface height modulations from the thermal TG of several nanometres, which paves the way to application of thermal TGs for synchrotron pulse selection in a broad energy range of ∼100 eV to 20 keV. Since the diffraction does not rely on a Bragg peak, the method can tolerate a finite bandwith, for example a pink beam from an undulator.

In the next section, we briefly review diffraction of hard X-ray beams from laser-generated thermal TGs. In Section 3 we discuss the synchrotron pulse picking scheme in detail and present experimental data, which is discussed in Section 4. Current limitations of our approach and an outlook based on theoretical calculations are presented in Section 5.

## 2. X-ray diffraction from high-amplitude thermal transient gratings   

**Figure 1:**
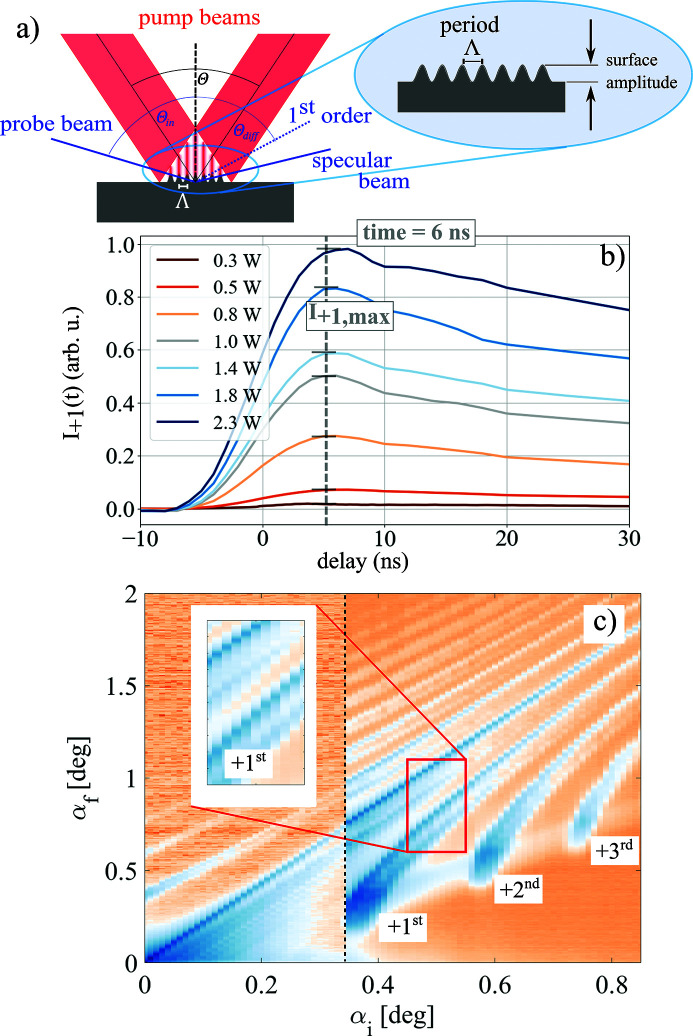
Experimental data. (*a*) Schematic of the optical generation and TRXRR probe of thermal TGs. (*b*) Measured first-order diffracted intensity from laser-generated thermal TGs. Measurements were performed at ID09 beamline at the European Synchrotron ESRF. The maximum of the diffracted intensity *I*_+1,max_ is reached at a delay of 6 ns (gray dashed line). (*c*) TRXXR measurement at a delay of 6 ns. The horizontal dashed line marks the concatenation of two measurements with different integration times of the detector. The measurement reveals multiple diffraction orders which are smeared out along α_f_ (*cf.* inset).

Laser-generated thermal TGs consist of a periodic modulation of the sample surface height due to absorption of optical energy and subsequent thermal expansion. The lateral modulation of the optical intensity, which leads to the periodic expansion profile, is generated by interfering two laser pulses on the sample surface, as shown in Fig. 1 ▸(*a*). All-optical generation and probing of TGs is extensively discussed in the literature for thermal gratings and surface acoustic waves (Rogers *et al.*, 2000 ▸), phonon-polaritons (Goldshteyn *et al.*, 2014 ▸), magnetoacoustics (Janušonis *et al.*, 2016*a* ▸,*b* ▸) and coherent four-wave-mixing measurements (Knoester & Mukamel, 1991 ▸). Only recently thermal TGs were investigated by diffracting hard X-ray pulses under grazing-incidence geometry (Sander *et al.*, 2017*a* ▸). Measurement of the transient surface excursion yields insights into the dynamics of coherent surface acoustic waves (Sander *et al.*, 2017*b* ▸) and of the lateral and perpendicular thermal diffusion (Pudell *et al.*, 2019 ▸). The diffracted X-ray intensity in different diffraction orders is directly linked to the amplitude of the surface modulation. For the *n*th diffraction order, the intensity reads
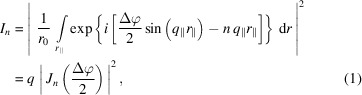
where *J*_*n*_ denotes the *n*th-order Bessel function of the first kind and 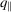 = 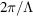 is the wavevector associated with the thermal TG. The phase term Δφ is a function of X-ray wavelength λ_Xray_, grating period Λ, incidence angle α_i_ and the surface amplitude *u*,
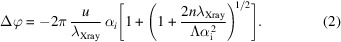
We have discussed the limits of the diffraction model [*cf.* equations (1) and (2)] in a previous article (Sander *et al.*, 2017*b* ▸). It is important to mention here that the maximum X-ray diffraction efficiency predicted by our model is 33% and that the necessary surface amplitude to reach this maximum value is a few nanometres.

Having connected the surface amplitude of the thermal TG to the diffracted intensity, one can now follow the amplitude decay over time due to thermal diffusion by time-resolved X-ray reflectivity (TRXRR) measurements. The analytical solution of the heat diffusion equation for a spatially periodic initial value problem allows for quick extraction of the lateral and perpendicular thermal conductivity from such measurements (Käding *et al.*, 1995 ▸). However, in this work we are interested in maximizing the diffracted X-ray intensity from a lateral thermal grating and do not retrieve thermal material properties from our measurements.

According to our diffraction model, maximizing the diffracted X-ray intensity requires high surface amplitudes of the thermal grating. However, energy absorption may lead to damage of the material if the sample temperature rises above the damage threshold. The peak temperature *T*_m_ generated by the excitation pulse can be estimated with the help of the diffusion parameter 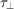 = 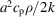, where *a*, *c*_p_ρ and *k* denote the optical penetration depth, volumetric specific heat and thermal conductivity, respectively. It is important to note that *T*_m_ is a function of the duration τ of the optical excitation pulse (Shayduk & Gaal, 2020 ▸). It can be approximated by
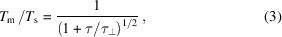
where *T*_s_ is the peak temperature generated with an ultrashort optical pulse (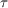 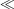 100 fs). A detailed derivation of equation (3) can be found elsewhere (Shayduk & Gaal, 2020 ▸). Our pulse picking scheme makes use of this effect: by employing an excitation pulse with a duration of 10 ns instead of only 1 ps we achieve a tenfold increase of the surface amplitude without inflicting any damage on our pulse picker sample.

Experimental data are shown in Fig. 1 ▸(*b*). The figure depicts the measured diffracted intensity in the +1st diffraction order *I*_+1_ over the pump–probe delay. To generate the TG in the sample we image a phase mask with *d* = 4 µm period on the sample surface with a magnification factor *M* = 1/2. Thus, the TG period was Λ = *d*/2*M* = 4 µm. The sample was a thin film heterostructure consisting of a top layer of optically transparent LaAlO_3_ (LAO), an optically opaque layer of La_0.7_Sr_0.3_MnO_3_ (LSMO) and a transparent NdGaO_3_ (NGO) substrate. The layer thickness was 100 nm and 65 nm, respectively. We have studied similar samples with short excitation pulses (Bojahr *et al.*, 2015 ▸; Sander *et al.*, 2017*a* ▸,*b* ▸, 2019 ▸) and compared the effect of short- and long-pulse excitation directly (Shayduk & Gaal, 2020 ▸). With equation (3) we find a diffusion parameter τ_⊥_ ≃ 320 ps. Due to the grazing-incidence angle of the X-ray probe pulse, the footprint of the pump and probe beams are larger than the sample surface, which makes it difficult to precisely determine the excitation fluence. We refrain from listing rough values for the excitation fluence and provide the laser output power as a measure for the excitation strength instead. We used a commercial amplified laser system (Coherent Legend) which delivers optical pulses at a wavelength of 800 nm, a repetition rate of 1 kHz and a pulse energy of 2 mJ. For the measurements shown in Fig. 1 ▸(*b*), we blocked the amplifier seed pulse to obtain a long pulse duration of 10 ns. The slow signal rise of the transients shown in Fig. 1 ▸(*b*) is evidence of the long excitation pulse. The diffracted intensity of all transients in Fig. 1 ▸(*b*) is normalized to the maximum value.

A time-resolved X-ray reflectivity (TRXRR) measurement at a pump–probe delay of 6 ns and a pump laser power of 2 W is depicted in Fig. 1 ▸(*c*). Due to strong diffuse scattering at grazing incidence angles, the data were recorded in two measurements with longer averaging at higher incidence angles. The concatenation of both measurements is marked by the horizontal black dashed line at an incidence angle of α_i_ = 0.35°. At the high surface amplitude of the transient grating we observe three positive diffraction orders and seven negative diffraction orders. Interestingly the diffracted intensity is smeared out along the exit angle α_f_. We highlight this feature in the magnification of the red square, which shows a pronounced double structure of the specular beam and of the first diffraction order, respectively.

The data shown in Fig. 1 ▸ were measured at the ID09 beamline at the European Synchrotron ESRF. X-rays were delivered by the U17 undulator and monochromated to a relative bandwidth Δ*E*/*E* = 10^−4^ at an energy of 15 keV. A detailed discussion of the experimental setup can be found elsewhere (Shayduk & Gaal, 2020 ▸; Sander *et al.*, 2017*a* ▸,*b* ▸). Here, we want to point out that the X-ray beam diameter at the sample position was 20 µm, which results in a footprint of 20 µm × 5730 µm at grazing incidence angles of 0.2°. The elongation in the diffraction plane was larger than the sample with a surface area of 5 mm × 5 mm. Hence, an estimation of the diffraction efficiency from the grating was not possible. For that, we resort to the *in situ* and nanodiffraction beamline P23 at PETRA III, DESY. Experiments at this facility are discussed in the next paragraph. In Section 4 we discuss the influence of the beam footprint and overlap with the excitation laser in detail and compare experimental data recorded at both facilities.

## 3. Synchrotron pulse picking using thermal transient gratings   

**Figure 2:**
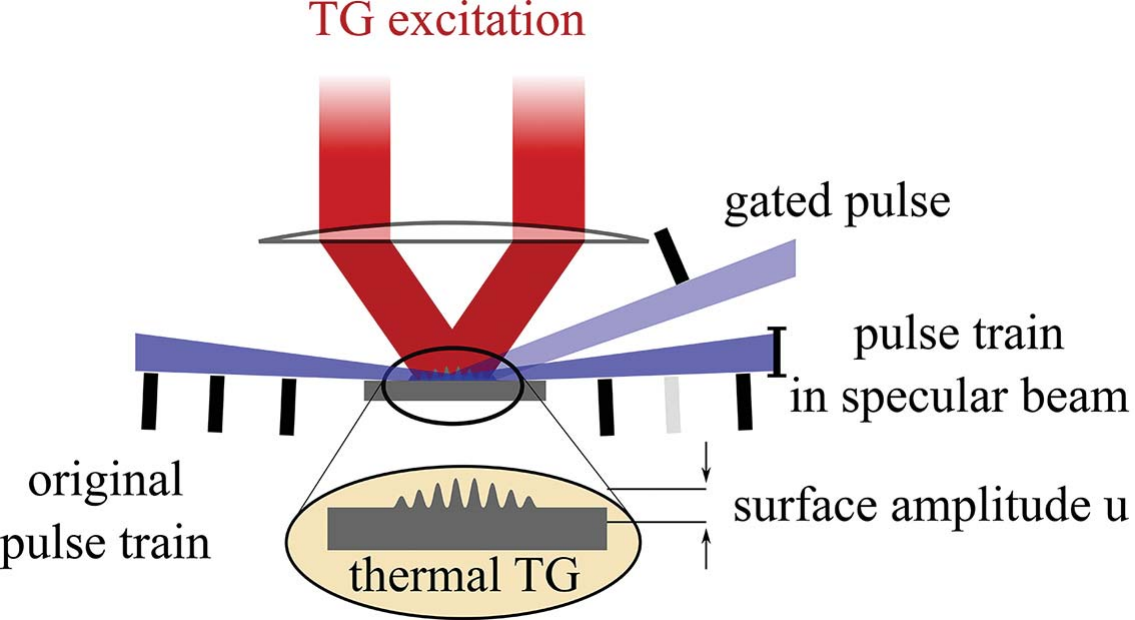
Pulse picking principle. X-ray pulses from the synchrotron impinge the NanoGate sample under grazing incidence and are diffracted away from the specular beam upon optical transient grating (TG) excitation. The main beam is blocked and only diffracted pulses are transferred to the sample.

In this section we demonstrate our new pulse picking approach by selecting a single X-ray pulse out of 255 consecutive synchrotron pulses. Our method is sketched in Fig. 2 ▸. X-ray pulses from a sychrotron storage ring impinge the sample at grazing incidence below the critical angle of total external reflection. Without TG excitation, the pulses are reflected in a specular beam. Upon inscribing a thermal TG, the X-ray pulse is diffracted into a higher order and can be separated by an aperture or by an analyzer crystal.

**Figure 3:**
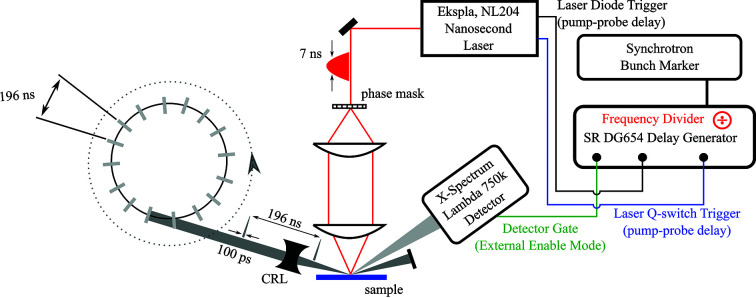
NanoGate layout. Experimental setup for NanoGate characterization measurements performed at P23 beamline at PETRA III, DESY. A Q-switched laser is synchronized to the PETRA III bunch clock and delivers optical excitation pulses with a duration of 7 ns to the TG setup (Pudell *et al.*, 2019 ▸) at a repetition rate of almost 1 kHz. The synchronization unit is also used to tune the pump–probe delay. The X-ray bunch spacing in 40 bunch mode is 196 ns and the X-ray pulse duration is 100 ps. Diffracted photons are detected by a hybrid pixel area detector in external gating mode (Pennicard *et al.*, 2013 ▸) (X-Spectrum LAMBDA 750k).

We have implemented this scheme at the *in situ* and nanodiffraction beamline P23 at PETRA III (DESY). The experimental setup is depicted in Fig. 3 ▸. Again we use an LAO/LSMO thin film heterostructure with thickness of 194 nm and 82 nm, respectively, grown on NGO substrate. The sample surface area was 10 mm × 10 mm to better shadow the direct beam at gracing incidence angles. Optical excitation pulses come from a Q-switched laser (Ekspla NL204) which delivers pulses with a duration Δ*T* = 7 ns at a wavelength of λ = 1064 nm and a pulse energy of 4 mJ. We use a delay generator (Stanford Research Systems DG645) which was synchronized to the synchrotron bunch marker to divide the bunch frequency to approximately 1 kHz and to generate trigger pulses for the laser and X-ray area detector. The pump–probe delay is implemented by scanning the laser diode and Q-switch trigger of the laser with 100 ps precision. Laser pulses are subsequently coupled into the TG setup which consists of a transmission phase mask and a system of lenses that images the phase mask onto the sample. The TG setup is discussed in detail elsewhere (Pudell *et al.*, 2019 ▸).

The P23 beamline delivers monochromatic X-ray pulses which can be focused to 1.6 µm × 200 µm (V × H) with a divergence of 0.5 mrad. In the horizontal direction the beam size was further reduced to 20 µm with a pair of slits. For our measurements the monochromator [Si(111) with Δ*E*/*E* = 10^−4^] was tuned to an energy of 10.2 keV. The synchrotron was operated in 40 bunch mode, *i.e.* the temporal gap between two consecutive X-ray pulses is 196 ns. Again we use a phase mask with a *d* = 4 µm period which was now imaged with a magnification factor *M* = 1 onto the sample. Thus, the laser-generated thermal TG at the surface had a periodicity of Λ = 2 µm, *i.e.* only half the period of the ID09 measurement. In this configuration the incident beam is diffracted to higher angles which reduces the X-ray footprint on the sample and facilitates separation of the diffracted intensity from the specular reflection. The measured 1/e decay time for the transient grating was 52 ns, *i.e.* the TG is almost diffused within the bunch spacing *dt* = 196 ns. However, for shorter bunch spacing the thermal grating can be removed with a second TG excitation as described elsewhere (Pudell *et al.*, 2019 ▸). The specular reflection from the sample was blocked and photons diffracted into higher orders were captured with a hybrid pixel area detector (Pennicard *et al.*, 2013 ▸) with a pixel size of 55 µm × 55 µm (X-Spectrum, Lambda 750k). The detector was used in an external enable mode for electronic gating (Ejdrup *et al.*, 2009 ▸; Shayduk *et al.*, 2017 ▸). The electronic gate was set by the delay generator unit to values between 100 ns to 50 µs.

The sample was mounted on a 5+2 circle diffractometer in grazing incidence geometry. The detector distance to the sample was 1 m. The sample was exposed to a constant flow of room-temperature nitrogen gas for cooling, which had a noticeable effect on the stability and thermalization of the sample after exposure to the laser. The average laser power impinging the sample during the measurements was approximately 2 W.

## 4. Results and discussion   

**Figure 4:**
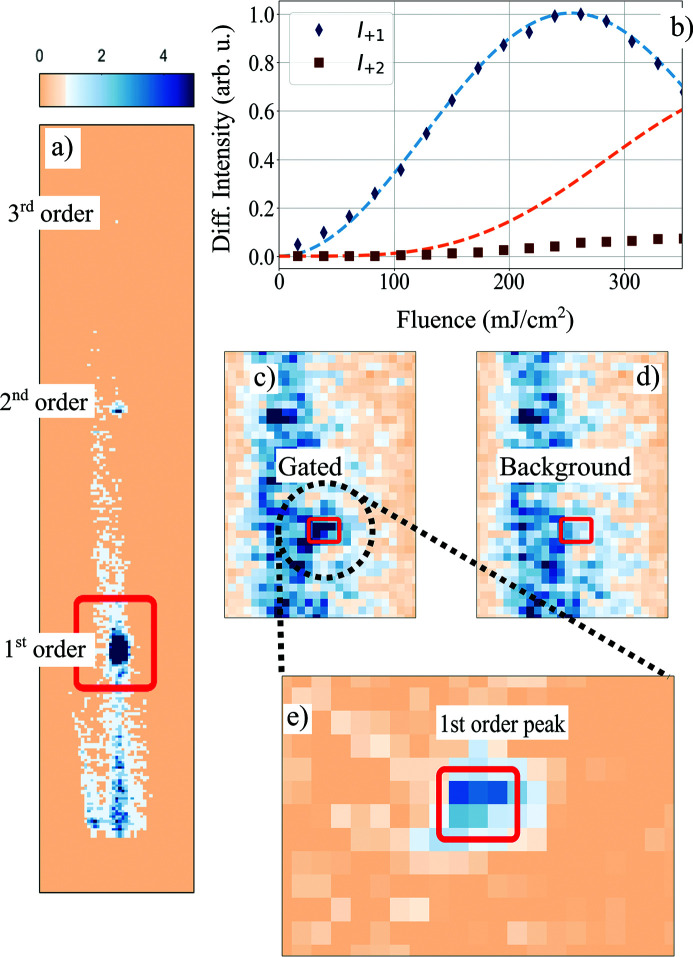
Synchrotron pulse gating. (*a*) Detector image depicting diffraction from a single synchrotron pulse in the first, second and third diffraction order. The pixel size is 55 µm × 55 µm, the red square marks the area (25 × 35 pixels) which is shown in (*c*) and (*d*). (*b*) Fluence dependence of the maximum diffracted intensity in the first (black diamonds) and second (brown squares) diffraction order. The dashed lines show calculated intensity using equation (1). The intensity of the first and the second order do not have the predicted ratio. (*c*, *d*) Diffraction in the first order with an external detector gate width of 50 µs, *i.e.* averaging 255 X-ray pulses from the synchrotron. Both images show the region marked with a red square in (*a*) and use the same colorscale. The long streak stems from diffuse surface scattering. The first diffraction order in the gated [(*c*)] and background [(*d*)] image is marked by the red rectangle. (*e*) Background-corrected image (20 × 12 pixels) of the first-order diffraction peak. The image was produced by subtracting the images (*c*) and (*d*).

Test results of our pulse picking method are depicted in Fig. 4 ▸. Panel (*a*) shows diffracted intensity of a single X-ray pulse from the thermal transient grating on the area detector. The detector gate width was set to 140 ns, *i.e.* shorter than the interval between two X-ray pulses. The specular beam is blocked and the high-intensity areas stem from diffraction in the first and second order. The colorbar was chosen to saturate high intensities in order to pronounce the diffuse background. The incidence angle was set to 0.28° and the direct beam was completely shadowed by the sample. The specular reflex is blocked in Fig 4 ▸(*a*) to avoid saturation of the detector.

In order to maximize the diffraction efficiency in the first order, we perform a fluence scan similar to Fig. 1 ▸(*b*). From the transient diffraction measurement we determined the delay of maximum diffraction from the thermal TG and subsequently scan the excitation fluence at that fixed delay. The result of that scan is shown in Fig. 4 ▸(*b*) for diffraction into the first (*I*_+1_) and second (*I*_+2_) orders. The maximum of *I*_+1_ is reached at a fluence of 250 mJ cm^−2^ and the fluence dependence follows the predicted Bessel-function from equation (1). However, comparison with the intensity of the direct beam yields a diffraction efficiency of only 0.1%, *i.e.* much lower than the expected 33% from our diffraction model. The dashed blue lines depict results of our diffraction model [*cf.* equation (1) and (2)] normalized to the measured *I*_+1_. The calculated intensity in the second order is significantly lower than the values, indicating deviations of the surface deformation from a pure sine modulation. We will discuss possible distortion mechanisms in more detail in the next section.

Now we determine the on–off contrast of our pulse picking scheme: we increase the detector gate width from 140 ns to 50 µs so that each image accumulates the intensity of 255 X-ray pulses with a spacing of 196 ns each. For the image shown in Fig. 4 ▸(*c*) a thermal TG was excited to select one of the 255 pulses incident on the sample. The image shown in Fig. 4 ▸(*d*) was accumulated over 255 pulses without exciting a thermal TG, *i.e.* all intensity stems from accumulated background. Both images show strong diffuse scattering and look similar at the first glance. Only a few pixels, which are marked by the red square, show a difference in intensity, which stems from the single selected pulse from the thermal TG. To remove the accumulated background, we subtract images (*c*) and (*d*). The result is shown in Fig. 4 ▸(*e*). The intensity of the selected pulse is 2.1 times the intensity of the accumulated background in the same pixel which corresponds to an on–off contrast of ∼550 (Sander *et al.*, 2016 ▸, 2019 ▸). If the theoretical diffraction efficiency of 33% were achieved, the on–off contrast would exceed 10^5^. In the next section, we will elucidate current limitations of the pulse picker and lay out improvements that enhance the performance to the theoretical optimum.

## 5. Current limitations and future improvements of the pulse picking scheme   

**Figure 5:**
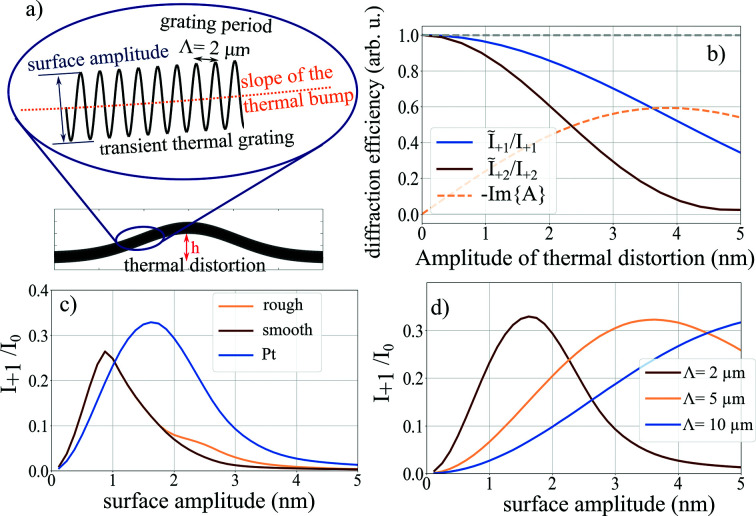
Optimization of the NanoGate performance. (*a*) Surface profile after TG excitation consisting of a long-range thermal distortion and a short-range thermal grating. (*b*) Theoretical diffraction efficiency from the surface profile shown in (*a*). (*c*) Diffracted intensity in the first order versus surface amplitude for different surface properties. (*d*) Diffraction efficiency versus surface amplitude for optimized structures at different TG periods.

Data measured at ID09, ESRF (not shown), and at P23, PETRA III [*cf.* Fig. 4 ▸], show significantly lower intensity in higher diffraction orders than expected. A reasonable explanation for this deviation is suggested by the splitting of the diffraction peaks that is highlighted in the inset of Fig. 1 ▸(*c*). The laser-induced thermal TG excitation of the sample leads to deformations on different length scales. First and foremost there is the expected surface modulation from the TG with a period of a few micrometres. The surface amplitude required to reach the maximum diffraction efficiency from the grating varies between 2 nm and 5 nm, depending on the actual experimental configuration (Sander *et al.*, 2017*b* ▸). Second, we also expect a long-range modulation of the surface on the length scale of the excitation area due to accumulated heat in the substrate (Shayduk & Gaal, 2020 ▸). The rising and falling slope of such deformations results in additional tilting of the diffraction geometry (Reinhardt *et al.*, 2016 ▸), thus generating the observed splitting of the diffraction peaks. The deformation amplitude *h* may be much larger than the thermal TG surface amplitude. An approximate sketch of such a total surface deformation is depicted in Fig. 5 ▸(*a*).

In order to estimate the effect of *h* on the diffraction efficiency in the +1st order, we include a Gaussian long-range surface modulation δ(*r*_∥_) with amplitude *h* in equation (1) resulting in
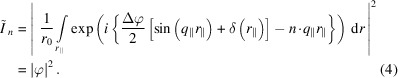
To evaluate the effect of δ(*r*_∥_) on the diffraction efficiency, we depict the ratio of the diffracted intensity from the distorted and undistorted surface 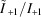 as a function of the distortion amplitude *h* by the blue line in Fig. 5 ▸(*b*). A similar ratio for the second-order diffraction 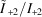 is depicted in the dark red line. The calculation assumes a grating period of 2 µm, an incidence angle α_i_ of 0.2° and a surface amplitude *u* of 3.12 nm. Already small long-range thermal deformations result in a fast drop in the diffracted intensity. It is apparent that the higher-order intensity drops more rapidly upon increase of *h*. The reason for the drop in intensity is a dephasing of the reflected beam across the excitation region, as shown by the oscillating imaginary part of the diffraction integral φ in equation (4) (light orange dashed line). We estimated the influence of the surface roughness on the diffracted intensity in a similar way by replacing the long-range distortion δ(*r*_∥_) with short-range random height fluctuations. The estimation predicts that realistic roughness values as measured on our samples with an atomic force microscope have no noticeable influence on the diffracted intensity.

Although equations (1) and (4) yield reasonable estimations of the diffracted intensity, the model is limited to diffraction below the critical angle of total reflection. For larger incidence angles, propagation effects in the medium may become important, which are not considered in the calculation of 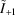. In order to better estimate the real diffraction efficiency and in order to find the best operation conditions for the pulse picker, we perform ray-tracing simulations of grazing incidence diffraction from sinusoidal surface deformations imprinted on our sample.

Figure 5 ▸(*c*) depicts the simulated diffracted intensity as a function of the deformation amplitude for a perfect and for a rough surface (light and dark red line) of the LAO/LSMO/DSO heterostructure sample. The X-ray energy in the simulation was 10.2 keV and the incidence angle was 0.15°. As expected from equation (4) there is only negligible influence of the surface roughness. However, the peak diffracted intensity is only 25% and therefore lower than the theoretical maximum. The efficiency may be improved by coating the sample with a dense material, *e.g.* platinum (Pt) (blue line). Here, the theoretical diffraction maximum is reached at a slightly higher surface amplitude of 1.65 nm. Finally, we compare the influence of the spatial periodicity Λ on the diffracted intensity *I*_+1_. Larger spatial periods require higher surface amplitudes to reach similar diffraction efficiency. In addition the angular separation of specular and first-order diffraction decreases, which makes it more difficult to separate the isolated diffracted pulse from the specular background. This behavior is also expected from our theoretical model [*cf.* equations (1) and (2)].

## 6. Conclusion   

In conclusion we have demonstrated a new method for selecting individual synchrotron X-ray pulses based on laser-induced thermal transient gratings. To achieve sufficient surface amplitudes, we employ nanosecond optical pump pulses. The optical excitation allows for controlling the surface deformation on timescales of the order of the excitation pulse. In first measurements we successfully demonstrated pulse gating although the theoretical limit of the diffraction efficiency was not reached. However, the switching contrast, which is the more challenging parameter for a pulse picker, is similar to alternative pulse picking schemes, provided the diffraction efficiency can be increased in future experiments.

Our simulations outline a pathway to an improved performance of the pulse picking scheme. A major challenge for the implementation of this method is avoiding the long range thermal distortion. This could be achieved by a combination of sample cooling and use of different substrate materials. Further improvements consist of depositing a high-density coating on the sample surface. Pt seems to be an adequate material which has already been investigated in strong optical pumping conditions (Shayduk *et al.*, 2016 ▸). Under optimal conditions we expect a diffraction efficiency from the grating of up to 30%. The pulse picker especially suits conditions at fourth-generation synchrotrons due to the small beam size, high collimation and relatively narrow spectral width.
